# InterTan nail versus Gamma3 nail for intramedullary nailing of unstable trochanteric fractures

**DOI:** 10.1186/s13000-014-0191-y

**Published:** 2014-10-01

**Authors:** Dankai Wu, Guangkai Ren, Chuangang Peng, Xuanlin Zheng, Fengmin Mao, Yueyang Zhang

**Affiliations:** Department of Orthopedics, Orthopeadic Hospital, Jilin University School of Second Hospital, 218 Ziqiang Road, Nanguan district, Changchun, Jilin 130041 China

**Keywords:** InterTan, Gamma3, Trochanteric fractures, Outcomes, Complications

## Abstract

**Background:**

Trochanteric fractures (TF) have become a major source of morbidity and mortality in elderly. We conducted this study to compare the outcomes of unstable trochanteric fractures treated with the InterTan nail and Gamma3 nail.

**Methods:**

Between January 2008 and May 2013, patients aged 60 years or older with a diagnosis of unstable TF treated with InterTan nail or Gamma3 nail were included. Patients treated with InterTan nail were pair-matched to patients treated with Gamma3 nail in a 1:2 ratio. Radiographs were obtained at 1, 3, 6, and 12 months follow-up, and all implant position changes, complications, fixation failures and functional scores were recorded.

**Results:**

Eighty-seven patients were included in the InterTan nail group, and 174 pair-matched patients were included in the Gamma3 nail group. Preoperative scores were similar between the 2 groups. There are significant improvements postoperatively in both groups. The incidence of cut-out and femoral shaft fracture were significantly higher in the Gamma3 nail group than the InterTan nail group (*P* = 0.024 and *P* = 0.044, respectively). Patients treated with the InterTan nail experienced longer fluoroscopy and operative times.

**Conclusions:**

The InterTan nail may have a tendency in better outcomes for patients with unstable TF. However, the limited period of follow-up and inherent defects of nonrandomized trials indicate that better-designed randomized controlled trials will be required.

**Virtual Slides:**

The virtual slide(s) for this article can be found here: http://www.diagnosticpathology.diagnomx.eu/vs/13000_2014_191

## Background

Trochanteric fractures (TF) are the second most common fractures of the proximal femur after femoral neck fractures and are major sources of morbidity and mortality in today’s ageing population [1,2]. Worldwide, the incidence of fractures of the proximal femur is increasing because of a demographic transition resulting in higher life expectancy [3-6]. To reduce the complications of prolonged immobilization, timely operative interventions providing sound stabilization of the fracture and early mobilization of the patients have become the preferred solution for the treatment of these fractures [7,8].

Once dynamic hip screw (DHS) internal fixation is one of the most primary options [9,10], but it performs less well with a relatively higher incidence of internal fixation failure for unstable TF. In addition, this surgical procedure may result in substantial blood loss, soft-tissue damage, and worsening of existing comorbidities in elderly patients [11]. Therefore, intramedullary fixation devices have become more popular due to biomechanical advantages in the treatment of unstable TF compared with DHS internal fixation.

The Gamma was designed for intramedullary fixation of highly volatile AO type 31-A2/A3 hip fractures [12]. However, there are some defects that exist in fixation of complex femoral fractures involving the potential secondary rotation of the head-neck fragment followed by collapse at the fracture site and cut-out. Now, Gamma 3 Locking Nail (GN) (Stryker GmbH & Co. KG, Duisburg, Germany) has been developed with a reduction of the diameter of the nail, a change to the valgus angle from 10 to 4 degrees, a change in the design of the femoral neck screw, and the possibility of dynamization [13]. The GN system can provide better clinical outcomes and a higher biomechanical stability compared with with older cephalomedullary devices [14].

The Intertan Nail (IN) (Smith & Nephew GmbH, Marl, Germany), using 2 cephalocervical screws in an integrated mechanism, shows increasing stability and resistance to intraoperative and postoperative femoral head rotation compared with the traditional intramedullary nailing system. A biomechanical study showed that the IN possesses more biomechanical benefits for internal fixation of unstable fractures compared with the traditional intramedullary nailing system [15]. Some studies reported that the surgical procedure had a good clinical outcome and a low number of complications [16,17].

The biomechanical study of Nüchtern et al. showed that IN achieves more stability with a higher tip apex distance and withstand the higher loads compared with GN [18]. However, no controlled trial has compared the clinical outcomes of the IN and GN. The primary aim of this study was to compare postoperative outcomes and complication rates between the IN and GN systems.

## Methods

This study was approved by our responsible Investigational Ethical Review Board. The study period was between January 2008 and May 2013. Each patient was prospectively enrolled in the study and was offered the choice of IN or GN system procedure. All patients presenting with symptomatic unstable TF of the femur (AO/ASIF classifications, 31-A2.1-3 and 31-A3.1-3) were proven by clinical examination, and plain radiographs. Informed consent was obtained from the patients or from family members if the patients were unable to consent. The advantages and defects of each procedure were discussed with the patients, and all questions were thoroughly answered.

The inclusion criteria for this study were patients aged 60 years or older, with a diagnosis of unstable TF caused by a low-energy trauma, treated with surgery of IN or the control GN procedure. We excluded patients with high energy trauma, open fractures, multiple fractures, pathologic fractures, American Society of Anesthesiologists (ASA) score of V, inability to work before injury and injured hip degenerative osteoarthritis before.

A total of 87 patients who were performed with IN system procedure for the treatment of TF met the inclusion criteria (InterTan group). A matched-pair group was created from a larger cohort of 524 patients undergoing GN system procedure during the study period (Gamma3 group). The matched-pair group was selected based on age within 2 years, gender, Body mass index (BMI), fracture type according to AO classification, and ASA score in respect of preoperative comorbidities. Both treatment groups were comparable in terms of general data preoperatively (Table 1).

**Table 1:** **Patient demographics**

**Factors**	**InterTan group**	**Gamma3 group**	***P*** **value**
Sample size (n)	87	174	NA
Age (mean + SD, years)	71.4 ± 9.7	72.6 ± 8.6	0.309
Gender (F/M)	67/20	131/43	0.759
Body mass index (kg/m^2^)	25.6 ± 3.7	24.8 ± 4.9	0.180
Side (left/right)	39/48	80/94	0.860
Smoking status	4	3	0.176
Detail of trauma, No.			
At home	58	119	
While walking	26	54	
Traffic accident	3	1	
AO classification (n)			0.237
A 2.1	32	60	
A 2.2	25	59	
A 2.3	15	27	
A 3.1	5	10	
A 3.2	4	11	
A 3.3	6	7	
ASA score (n)			0.168
1	13	25	
2	29	59	
3	37	70	
4	8	20	
Preoperative HHS (mean ± SD)	55.3 ± 8.6	56.7 ± 7.8	0.187
Preoperative NAHS (mean ± SD)	53.6 ± 7.8	55.2 ± 8.1	0.128

ASA, American Society of Anesthesiologists; HHS, Harris Hip Score; NAHS, the Non-Arthritic Hip Score; NA, not available; AO, SD, F/M.

Similar preoperative treatment, including a combination of skin traction, prophylactic medication for deep venous thrombosis and surgical infection and adequate pain relief, was performed for all patients. General anesthesia was used for all surgeries. Two hip surgeons performed all operations.

Surgery was conducted according to the standard protocols for the InterTan and Gamma3 nails, which are recommended by the manufacturer and have been described in earlier studies [16,19]. The IN is a solid titanium nail of 180 mm in length with a trapezoidal proximal end and has a diameter decreasing from 15.25*16.25 mm at the proximal end to 11 mm at the distal end. The proximal end of the nail will accept 2 cephalocervical screws: a larger superior 11-mm lag screw and a smaller 7-mm compression screw. The smaller screw is integrated into the larger and has the effect of creating an oval screw with a composite diameter of 15.5 mm. The GN used in the current study is a solid titanium nail with a diameter of 11 mm at the distal end, a length of 170/180 mm, and a lag screw angle of 125° or 130°. Both nails were inserted using a percutaneous technique.

A standard postoperative protocol was used for all patients. Suction drains were placed for 48 hours and prophylactic antibiotics were given for 24 hours postoperatively. Continuous passive motion was required twice daily after the drainage tubes were removed. All patients underwent plain anteroposterior and lateral radiographs on postoperative day 1, and the films were analyzed for fracture reduction and implant position. All patients were mobilized out of bed and started on a physical therapy program of weight-bearing as tolerated within the first few days. The patients were allowed to walk with non-weight bearing postoperatively week 4 and then take activities with partial weight-bearing 12 weeks after operation, according to the growth condition of callus assessed by X ray. Weight-bearing was not permitted until the fracture line was blurred.

Prior to the surgery and follow-up visits at 1 months, 3 months, 6 months, and 1 year after surgery anteroposterior and lateral views were acquired. All implant position changes, fixation failures, and complications were recorded. Additionally, hip range of motion; pain of affected limb; walking ability score; postoperative complications; Harris Hip Score (HHS); and the Non-Arthritic Hip Score (NAHS) were recorded.

Statistical analyses were performed using SPSS version 17.0 software. The 2-tailed, unpaired *t* test was used to evaluate differences between two groups, and the 2-tailed, paired *t* test was used to detect changes in preoperative to postoperative outcome scores. All continuous data were expressed as mean ± standard deviation (SD). For continuous variables, such as age, length of stay, blood loss, and operative time, independent sample *t* tests were used. Categorical variables were analyzed with the chi-square test. *P* < 0.05 was considered significant for all statistical analyses.

## Results

Patient’s demography and preoperative subjective and clinical parameters are shown in Table 1. No significant differences were found between the two groups, in terms of gender, age, BMI, smoking status, side of fracture, type of fracture and ASA score. At the final follow-up, none was lost in this study. Intraoperative data are shown in Table 2. Mean operative (*P* = 0.011) and fluoroscopy (*P* = 0.012) time were significantly longer in the InterTan group than the Gamma3 group. Regarding to blood loss, hospital stay, quality of reduction, and position of the distal end of implant, no significant differences were observed.

**Table 2:** **Intraoperative data**

**Variables**	**InterTan group**	**Gamma3 group**	***P*** **value**
Mean operative time (min)	63.7 ± 10.4	59.9 ± 11.8	0.011
Mean blood loss (mL)	87 ± 5	86 ± 6	0.181
Mean hospital stay (d)	10.83 ± 1.41	11.13 ± 1.25	0.081
Fluoroscopy time (min)	2.9 ± 0.16	2.6 ± 0.18	0.012
Reduction results (n)			
Anatomical	69	148	0.242
Acceptable	14	20	0.298
poor	4	6	0.648
Position of the distal end of implant (n)			
Medial	1	2	0.996
Central	82	163	0.855
Lateral	4	7	0.850

All complications for InterTan and Gamma3 groups are shown in Table 3. A total of 57 complications occurred: 16 in the InterTan group and 41 in the Gamma3 group (*P* = 0.394). Femoral shaft fractures were recorded in 11 patient (1 in in the InterTan group and 10 in the Gamma3 group), and better result was shown in the InterTan group (*P* = 0.044). In respect to cut-out rates, the IN system was superior as compared with the GN system (*P* = 0.024). This suggested that IN system may get the edge in cut-out and femoral shaft fractures. With regard to intraoperative complications, including bleeding, technical problems, surgical procedure changes, and other postoperative complications, the results in both the InterTan and the Gamma3 group were equal.

**Table 3:** **List of all complications for both InterTan and Gamma3 groups**

**Complications**	**InterTan (n = 87)**	**Gamma3 (n = 174)**	***P*** **value**
Total	16	41	0.394
Intraoperative complication	2	4	1.000
Bleeding	2	4	1.000
Technical problem	0	1	0.479
Surgical procedure change	0	0	NA
Postoperative complication	14	37	0.320
Implant loosening	0	2	0.315
Cutout	1	14	0.024
Implant breakage	0	0	NA
Femoral shaft fracture	1	10	0.044
Nonunion	1	5	0.381
Superficial wound infection	0	0	NA
Wound hematoma	1	0	0.157
Hip pain	2	3	0.749
Thigh pain	2	4	1.000
Femoral neck shortening (mm)	2.2 ± 0.54	2.6 ± 0.31	0.648
Cardiovascular disorder	5	7	0.531
Deep venous thrombosis	5	8	0.695
Pulmonary embolism	0	0	NA
Urinary tract infection	4	7	0.828
Reoperation	2	5	0.786

Both the IN and GN patients reported higher scores in functional outcomes postoperatively than preoperatively. Table 4 shows the clinical and functional outcomes at the final follow-up. The mean HHS was 88.2 for the InterTan group and 85.6 for the Gamma 3 group. Statistical analysis of the two treatment groups revealed no significant difference (*P* = 0.076). Similarly, with respect to NAHS, hip range of motion and walking ability score, no significant differences were found.

**Table 4:** **Clinical and functional outcomes at the final follow-up**

**Outcomes**	**InterTan (mean ± SD)**	**Gamma3 (mean ± SD)**	***P*** **value**
Harris hip score			
1 year	88.2 ± 15.6	85.6 ± 14.9	0.076
The Non-arthritic hip score			
1 year	85.8 ± 13.2	84.7 ± 14.6	0.206
Hip range of motion, deg			
1 year	96.7 ± 17.6	94.9 ± 16.8	0.968
Walking ability score			
1 year	6.9 ± 1.9	6.2 ± 1.6	0.846

Deg: degrees;

SD.

## Discussion

To our knowledge, this is the first prospective study directly comparing the IN with GN procedures. The results of this study revealed longer operative time, shorter fluoroscopy time, and less incidence of cutout and femoral shaft fracture in the IN group. This matched-pair comparison suggested similar functional outcomes between two groups at 1-year follow-up. And the results with the InterTan and Gamma3 nails for the treatment of unstable proximal femoral fractures showed no important differences in terms of pains and most complicates rates.

TF are common fragility fractures in the elderly. There is a controversial issue about the optimal choice for the stabilisation of trochanteric fractures because there is still a lack of evidence for the use of intramedullary devices. Treating unstable femoral neck fractures with internal fixation is associated with various complications [20]. Previous studies have shown good results with the GN [14,21]. However, poor cut-out and the presence of femoral shaft fracture were reported in other studies [22,23]. The IN system has become increasingly popular with the development of innovative systems, which minimize incidence of fractures of distal femur and increase stability [24]. The IN with two integrated cephalocervical screws was introduced in 2005 for treating TF [20], and the results with this device were favorable in TF [16]. Nonetheless, there are still some defects in the IN system, such as higher costs and the need of operative skills.

In this study, a longer mean operative time was shown in the IN group compared to the GN group. And both the operative times were longer than some previous studies [12,16]. This may be associated with different fracture types. In this study, the patients with unstable fracture were included, and therefore the fractures were more difficult to reduce. In addition, The IN is more difficult to insert into a poorly reduced marrow cavity due to a trapezoidal proximal end of the nail. Repeated reduction and manipulation, especially in more unstable fracture types, will result in longer operative and fluoroscopy time and more intraoperative blood loss [12,17].

Cutting out is a familiar problem in the osteosynthesis of trochanteric femoral fractures [14]. Cut-out rates, including the Z effect, have been reported to range from 3% to 10% with the GN [25-28]. In study of Vaquero et al. [12], they found no statistical difference in the cut-out rates between the proximal femoral nail antirotation (PFNA) and the Gamma3. They believed that cut-out appeared to result from poor positioning of the screw rather than being implant-related and the key of less cut-out was to make sure the proper position of the screw and the correct tip-to-apex distance [29]. However, no significant difference in position of implant and reduction results were shown in this study, but better result was demonstrated in cut-out of the IN group. In addition, there was a tendency for more IN patients to return to full weight bearing than GN patients. Unstable TF of femur treated with an intramedullary device are commonly related to mild pain at the side of the fracture and in the middle thigh [30]. The results of this study suggested the design of the implant may still be one of effect factors result in cut-out.

The GN group presented a higher incidence of femoral shaft fracture in this study. However, Yao et al. reported that femoral shaft fractures were observed in 6 of the 107 patients with intertrochanteric fractures in their study and found no significant difference between the GN group and control group [21]. The diameter of the IN tapers from 13.5 mm in the middle to 11 mm at the tip, which has a stress dispersion effect on the IN and inner cortex and avoids stress overconcentration around the nail tip [16]. However, the design of the GN is short of the advantage of IN system and therefore easier to lead to the femoral shaft fracture. Moreover, ensuring sufficient reaming of the intramedullary cavity can reduce the incidence of intraoperative femoral shaft fractures [31].

Some limitations remained in this study. First, the follow-up period was relatively short. The long-term effect of the two surgical managements was unable to find out. The second limitation was that this study was not a randomized trial. The results could not adequately evaluate the outcomes of two surgical methods. Third, the sample size of total the patients is relatively small, which may affect the power in statistical analysis. In future, in order to better evaluate the outcomes of the two treatment strategies, a well-designed randomized controlled trial (RCT) will be needed.

## Conclusion

In this study, postoperative recovery in terms of functional scores, hospital stay and the incidence of total complications is equal in both IN group and GN group. However, the incidence of cut-out and femoral shaft fracture were decreased in IN group comparing with GN group. The IN may result in better outcomes for patients with unstable TF. For all that, future well-designed RCTs are needed to confirm the gold standard protocol for the treatment of unstable TF.
